# A Circuit Model for Working Memory Based on Hybrid Positive and Negative-Derivative Feedback Mechanism

**DOI:** 10.3390/brainsci12050547

**Published:** 2022-04-26

**Authors:** Hui Wei, Xiao Jin, Zihao Su

**Affiliations:** 1 Laboratory of Cognitive Model and Algorithm, Department of Computer Science, Fudan University, No. 825 Zhangheng Road, Shanghai 201203, China; 20210240323@fudan.edu.cn (X.J.); 012021129@fudan.edu.cn (Z.S.); 2 Shanghai Key Laboratory of Data Science, No. 220 Handan Road, Shanghai 200433, China

**Keywords:** working memory, neural network, computational model, hybrid positive and negative-derivative feedback, memory forgetting

## Abstract

Working memory (WM) plays an important role in cognitive activity. The WM system is used to temporarily store information in learning and decision-making. WM always functions in many aspects of daily life, such as the short-term memory of words, cell phone verification codes, and cell phone numbers. In young adults, studies have shown that a central memory store is limited to three to five meaningful items. Little is known about how WM functions at the microscopic neural level, but appropriate neural network computational models can help us gain a better understanding of it. In this study, we attempt to design a microscopic neural network model to explain the internal mechanism of WM. The performance of existing positive feedback models depends on the parameters of a synapse. We use a negative-derivative feedback mechanism to counteract the drift in persistent activity, making the hybrid positive and negative-derivative feedback (HPNF) model more robust to common disturbances. To fulfill the mechanism of WM at the neural circuit level, we construct two main neural networks based on the HPNF model: a memory-storage sub-network (the memory-storage sub-network is composed of several sets of neurons, so we call it “SET network”, or “SET” for short) with positive feedback and negative-derivative feedback and a storage distribution network (SDN) designed by combining SET for memory item storage and memory updating. The SET network is a neural information self-sustaining mechanism, which is robust to common disturbances; the SDN constructs a storage distribution network at the neural circuit level; the experimental results show that our network can fulfill the storage, association, updating, and forgetting of information at the level of neural circuits, and it can work in different individuals with little change in parameters.

## 1. Introduction

Working memory (WM) has the ability to store information “online” during cognitive processing, forming the essential foundation of cognitive activity. WM is an indispensable part of working and daily living [1,2,3,4,5]. The construction of a storage system of working memory needs to pay attention to four characteristics [6]. First, the storage system should have states that can persist briefly over time; second, it should contain sufficient capacity; and third, the states should be robust to noise, while the stored memory should not be severely disturbed. Finally, the stored memory should be able to be retrieved correctly if a relevant hint is given.

There have been many studies on WM. Compte et al. [7] created the recurrent network model to sustain the persistent neural activity associated with WM. They proposed a “ring” model in which neurons of the model cover all the angles uniformly along a circle. Therefore, the cells are spatially distributed on a ring. This model achieved notable success in simulating persistent activity related to visuospatial WM (vs. WM). However, little attention has been paid to the heterogeneities in network topology and long-range connections. Rolls et al. [8] proposed a model that can hold multiple items in its memory. They emphasized the importance of inhibitory synaptics in the success of the model. However, the parameters of the model need perfect tuning, which means the parameters of this model do not well simulate the ordering of memory in the human brain, so it cannot remember the order of memory. Additionally, the model does not have the ability to update WM. Kriete et al. [9] proposed a variable binding model. When we are faced with arbitrary instructions, we are able to understand and follow them with remarkable ease. It has been argued that this ability is related closely related to symbol processing, which depends critically on the ability to represent variables and binding them to arbitrary values. In the variable binding model, a given population of neurons is assumed to encode a particular type of information, and different patterns of activity correspond to different possible contents of each type. This model can save a pattern’s encoding and its location in a different part of the prefrontal cortex. They used the variable binding mechanism to increase the flexibility of the network, but they did not describe how the pattern and location bound together or how to update memory when the WM was full. Furthermore, these models have strict requirements on the parameters of the network, which means a slight change will have a great impact on network performance.

Cortical neurons receive massive amounts of excitation and inhibition [10,11,12,13,14], and the pathways between pyramidal cells have slower kinetics than the pathways from pyramidal cells to interneurons [15,16,17]. The negative-derivative feedback mechanism can inhibit pyramidal cells and interneurons. Hence, it can counteract drift in persistent neural activity [18]. There has been some research on working memory regarding how interacting neurons implement the basic components of cognition; integrating these basic components to simulate more complex cognitive processes has become equally urgent and important [19]. Allen Newell believes that cognitive hypothesis testing needs to be supplemented by building a comprehensive computational model of task execution [20,21,22]. We try to describe the meaning of our work by comparing the process of memory management in computer operating systems with the process of working memory in the human brain (Table 1).

## 2. Materials and Methods

### 2.1. Hybrid Positive and Negative-Derivative Feedback

**Figure 1:**
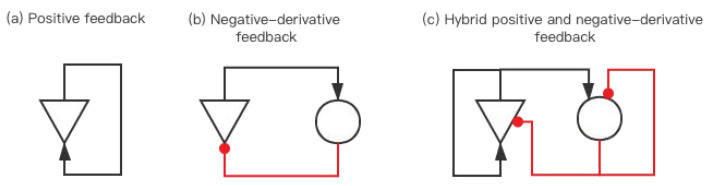
Comparison of different models. The solid red line is an inhibitory connection, and the solid black line is an excitatory connection. (**a**) The positive feedback model can maintain the continuous activity of neurons. (**b**) The negative-derivative feedback model can offset the drift of the neuron state to a certain extent. (**c**) Hybrid positive and negative-derivative feedback model (HPNF).

The essence of WM is the temporary sustaining of information, which is achieved by cortical neural circuits and requires the basic functional units of cortical neurons [26]. From a biophysical point of view, individual neurons are inherently “forgetful” as a result of the rapid leakage of electrical current from the cell membrane. If a memory storage unit includes a circuit of positive feedback, this circuit can accurately replace the leakage current (Figure 1a). In theory, this activity could continue indefinitely. However, if the synaptic strength of the positive feedback is too strong or too weak, neuronal activity rises or falls rapidly until it saturates or quiets at a baseline level (resting state). Therefore, the positive feedback model [18] needs to accurately adjust the level of feedback and maintain the sensitivity to common disturbances.

According to the positive feedback model in Figure 1a, a single neuron cannot sustain information in a short period of time. The latest research on the frontal cortex circuit reports the difference in the dynamics of excitatory pathways between excitatory and inhibitory neurons [18,27,28]. The excitatory-to-excitatory connection is usually associated with positive feedback and has relatively slow dynamics due to a large amount of slow NMDA (N-methyl-D-aspartic acid) conduction. The excitatory-to-inhibitory connection is necessary to drive negative feedback, and it is relatively fast. The characteristics of these two connections naturally lead to a corrective, negative-derivative form of feedback that counteracts drift in sustained activity [18] (Figure 1b). Negative-derivative feedback can complement positive feedback by the opposite drift caused by the imperfect adjustment of the positive feedback (Figure 1c). We use mathematical models to explain.

#### 2.1.1. Positive Feedback

This equation describes the positive-feedback model, where τ denotes the intrinsic time constant that receives a transient input *I*(*t*) to be stored in memory. *Wpos* is the synaptic strength of positive feedback, and the memory storage unit should exhibit only very slow changes dr in its firing rate *r*. The positive-feedback model maintains persistent firing with a continuous feedback current. The model becomes balanced when the feedback is just right (*Wpos* = 1). However, if the feedback is too low (*Wpos* < 1), the memory activity decays to a baseline level. Similarly, if the feedback is too large (*Wpos* > 1), the memory activity grows exponentially on a timescale set by the intrinsic time constant τ [18].

(1) $$\tau \frac{dr}{dt}=-\left(1-W_{pos}\right)r+I\left(t\right).$$

#### 2.1.2. Negative-Derivative Feedback

**Figure 2:**
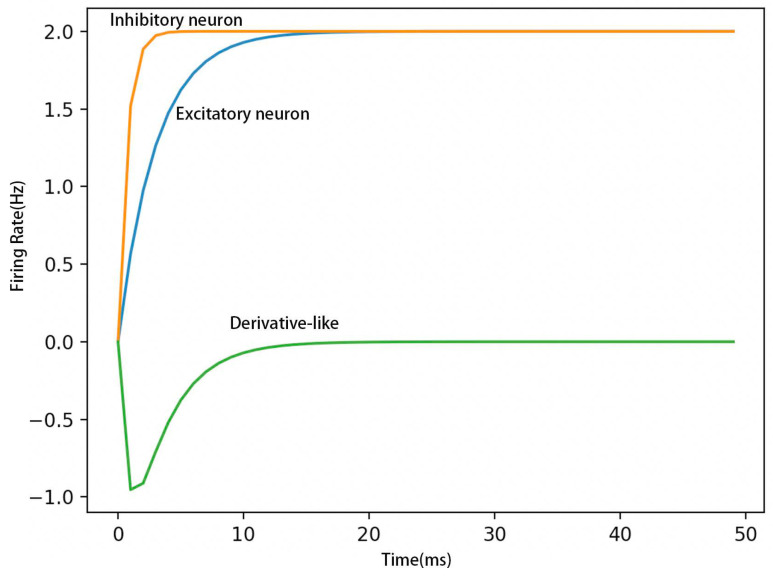
Firing rate experiment of negative-derivative feedback.

Equations (2) and (3) describe the negative-derivative feedback model, where τ denotes the intrinsic time constant that receives a transient input *I*(*t*) to be stored in memory. Wpos is the synaptic strength of positive feedback, Wder is the synaptic strength of negative feedback, $r_{E}$ is the firing rate of excitatory neurons, $r_{I}$ is the firing rate of inhibitory neurons. The negative-derivative feedback mechanism is based on the fact that excitatory and inhibitory inputs are equal in aggregates in cortical neurons and that pyramidal-to-pyramid and pyramidal-to-interneuron connections have different dynamics. Using Equations (2) and (3), and performing a simple subtraction, we obtain Figure 2, which describes why we call Figure 1b a negative-derivative feedback.

(2) $$\tau \frac{dr_{E}}{dt}=-W_{pos}r_{E}+I\left(t\right).$$

(3) $$\tau \frac{dr_{I}}{dt}=-W_{der}r_{I}+I\left(t\right).$$

#### 2.1.3. Hybrid Positive and Negative-Derivative Feedback

Considering the simple mathematical model described above, in order to successfully remember the offset after this input, the memory storage unit should only show a very slow change *dr*/*dt* in its firing rate *r*(*t*). This requires a positive feedback of the synaptic strength Wpos and a negative-derivative feedback of the synaptic strength Wder to counteract its inherent leakage of current. Combined with the positive feedback model, we obtain the following formula:

(4) $$\tau \frac{dr}{dt}=-r+W_{pos}r-W_{der}\frac{dr}{dt}+I\left(t\right).$$

We use positive feedback and negative-derivative feedback together to construct a hybrid positive and negative-derivative feedback (HPNF) model. The HPNF model solves the problem of poor stability in the positive feedback model. The HPNF model can resolve the problem of drift and leakage in neural activity. Positive feedback can offset the leakage of currents, and negative-derivative feedback can counteract drift in persisting activity caused by the deviation of positive feedback (Figure 1c). To remember the input, the memory storage unit should exhibit only very slow changes in its firing rate, which requires intrinsic leakage of current [18]. The HPNF model can maintain neural activity for a while because it is robust to common perturbations.

Next, we design a more robust network based on the HPNF model. We use neurons and the neural circuits they constitute to fulfill the self-sustaining information [29,30]. Then, we use the HPNF model to construct a memory-storage sub-network (SET).

### 2.2. Memory-Storage Sub-Network Based on HPNF Model—SET Network

**Figure 3:**
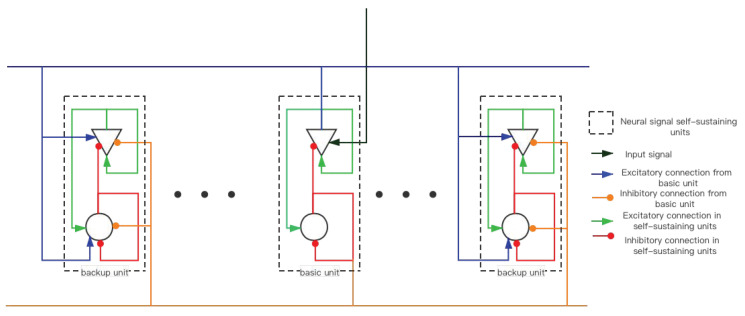
The SET network uses an HPNF model as a neural signal self-sustaining unit (NSSU). Triangles represent excitatory neurons (pyramidal cells, etc.) and circles represent inhibitory neurons (interneurons, etc.). The NSSU is divided into two types: basic unit and backup unit. The topology of the basic unit (backup unit) is identical to that of Figure 1c (HPNF). The difference between the basic unit and the backup unit is that the content stored in the backup unit is the backup in the basic unit.

The SET network is used to implement the self-sustaining mechanism of neural information, which is connected by multiple HPNF networks (Figure 3). Furthermore, it uses the neural information self-sustaining mechanism to achieve short-term storage of WM. In order to clearly explain the choice of the number of HPNF networks and the reason for the choice, we use the mean field model as the computational model to calculate the mean firing rate of the neuron population, and we conduct a series of experiments in Section 2.2.2.

Based on the research of Rombouts et al., we can make neurons learn to linearly store task-related information. During the learning process, these neurons are associated with different characteristics of an object and represent them with continuous activity during the memory delay [31]. Therefore, we can use the firing rate to express WM, such as in the mean field model. As a result, our model can use different units to process different characteristics of objects. Similarly, visual signals and auditory signals correspond to different processing circuits. This fits Baddeley’s WM model [32].

#### 2.2.1. Mean Field Model

We use the mean field model to describe the mean firing rate of one pyramidal cell group or one interneuron group. In the firing-rate model, *E* represents pyramidal cells, *I* represents intermediate neurons, $r_{E}$ and $r_{I}$ denote the mean firing rates of the element *E* and element *I*, and the synaptic state variables $s_{ij}$ denote the connection from element *i* to element *j*. These firing rate and synaptic state variables are governed by the below equations:

(5) $$\tau_{E}\frac{dr_{E}}{dt}=-r_{E}+f\left(J_{EE}s_{EE}-J_{EI}s_{EI}+J_{EO}i\left(t\right)\right)$$

(6) $$\tau_{I}\frac{dr_{I}}{dt}=-r_{I}+f\left(J_{IE}s_{IE}-J_{II}s_{II}\right)$$

(7) $$\tau_{ij}\frac{ds_{ij}}{dt}=-s_{ij}+r_{j},for\;i,j=E\;or\;I$$

where $\tau_{i}$ denotes the element *i*’s intrinsic time constant, $J_{ij}$ represents the synaptic connectivity strength from element *i* to element *j*, *i*(*t*) denotes the external stimulus current and noise, and *f(x)* represents the steady-state neuronal response to input current *x* and has the following Naka–Rushton form [33]:

(8) $$f\left(x\right)=M\frac{{\left(x-x_{\theta }\right)}^{2}}{x_{0}^{2}+{\left(x-x_{0}\right)}^{2}}h\left(x-x_{\theta }\right)$$

where *M* represents the maximal neuronal response, *x*θ denotes the input threshold, $x_{0}$ denotes the half-activation parameter, and *h(x)* represents the Heaviside step function. Throughout our study, the maximal response M = 100, the input threshold, $x_{\theta }\;=\;30$, and the half-activation parameter $x_{0}=30$. The parameters for the time constant are assigned as follows: $\tau_{E}$ = 20 ms, $\tau_{I}$ = 10 ms, $\tau_{EE}$ = 100 ms, $\tau_{EI}$ = 25 ms, and $\tau_{IE}$ = $\tau_{II}$ = 10 ms. The parameters for the synaptic connectivity are assigned as follows: $J_{EE}$ = 300, $J_{EI}$ = 450, $J_{IE}$ = 900, $J_{II}$ = 900, when *x* = *y*, it represents a connection inside the HPNF model; $J_{EE}$= 150, $J_{EI}$ = 300, $J_{IE}$ = 600, $J_{II}\;=\;600$, when x≠y it represents a connection between different HPNF models; other $J_{EO}$ = 0–6000.

#### 2.2.2. Reasons for Choosing the Number of NSSU (NEURAL Signal Self-Sustaining Unit)

The SET network uses an HPNF model as its NSSU. We use the mean field model to simulate the average firing rate of the SET network. After a series of experiments intended to compare eleven combinations of NSSUs, we find that the combination of eight NSSUs is the optimal choice.

**Figure 4:**
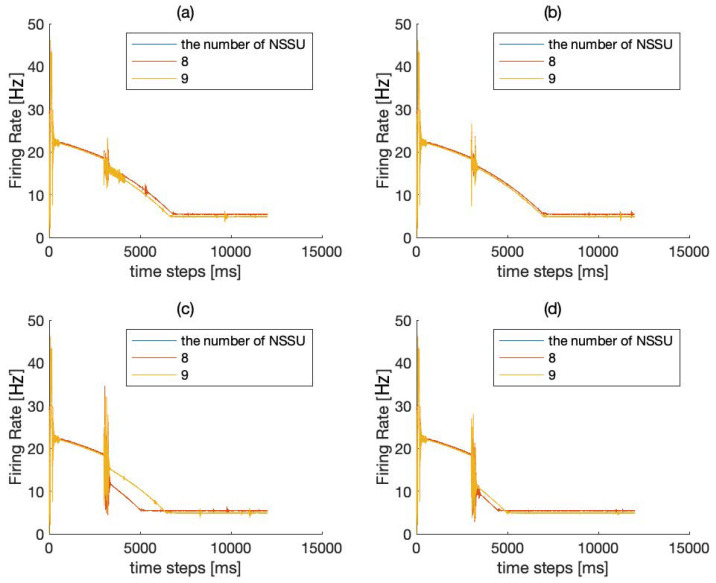
(**a**) Firing rate of 8–9 NSSUs when adding a maximum intensity of 5% $J_{EE}$ disturbance within a time range of 2%. (**b**) Firing rate of 8–9 NSSUs when adding a maximum intensity of 5% $J_{IE}$ disturbance within a time range of 2%. (**c**) Firing rate of 8–9 NSSUs when adding a maximum intensity of 5% $J_{EI}$ disturbance within a time range of 2%. (**d**) Firing rate of 8–9 NSSUs when adding a maximum intensity of 5% $J_{II}$ disturbance within a time range of 2%. The robustness of 8 NSSUs and 9 NSSUs is basically the same, but the computational complexity of 9 NSSUs is higher. This figure demonstrates that 8 NSSUs are the most suitable for this model.

**Figure 5:**
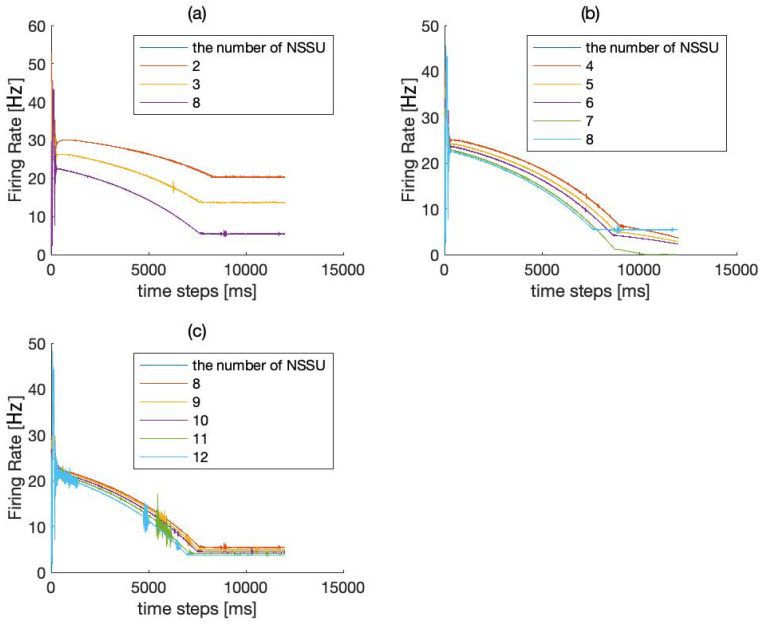
(**a**) Comparison of 2–12 NSSUs. The meaning of “time steps”: steps represent the number of iteration steps of the ordinary differential equation in the mean field model. The firing rate is related to time, so we call it “time steps”. After many experiments on the computer, we obtained the optimal number of NSSUs that can be included in the SET. Compared with the combination of 8 NSSUs, the average firing rates of the combination of 2–3 NSSUs show a steady trend. (**b**) When the number of NSSUs increases to 4–8, the average firing rates will not stabilize until the number of NSSUs increases to eight. (**c**) The firing rate of 8–12 NSSUs. The firing rates show a steady trend. However, the differences among them in the dynamic equation are very small.

In Figure 4, we measure the robustness of eight or nine NSSUs to noise interference. As shown in Figure 4 and Figure 5, it is more appropriate for the model to select eight NSSUs to form the entire memory circuit because the combination of eight NSSUs is the most stable. When the number of NSSUs is less than eight, the entire model will not stabilize in the end (Figure 5); when the number of NSSUs is greater than eight, the robustness of the entire model is not significantly improved (Figure 4), but the computational complexity will rise sharply. The SET network is composed of eight NSSUs. The fifth NSSU is called the basic unit, and the rest are called the backup units. The NSSUs can fulfill the storage of memory. The parallel connection of multiple NSSUs increases the robustness of the model and conducts redundant backup on the memory signals. The parallel connection of eight NSSUs is the most efficient.

### 2.3. Storage Distribution Network (SDN)

In Section 2.1 and Section 2.2, we describe the structure of the HPNF model and SET. In order to fulfill the storage and distribution process of memory items, we construct a storage distribution network (SDN) at the neural network level based on SET. SDN provides a possible explanation for the forgetting of WM at the microscopic neural network level.

#### 2.3.1. FIFO Mechanism

It is believed that the forgetting of the working memory in the human brain is due to “interference” [34]. In other words, the new memory covers the weaker old memory. However, we know very little about the detailed implementation of the mechanism at the neural network level. We try to use the FIFO mechanism to explain the forgetting of working memory. First input first output (FIFO) is the first in first out queue. We use the idea of FIFO to store memory items in order. The first incoming storage signal is the first to become weak and be replaced. We built SDN, which uses SET composed of pyramidal cells and interneurons as the “container” for storing memory. SDN follows the FIFO principle.

#### 2.3.2. Implementation of Storage Distribution Network at the Neural Circuit Level

**Figure 6:**
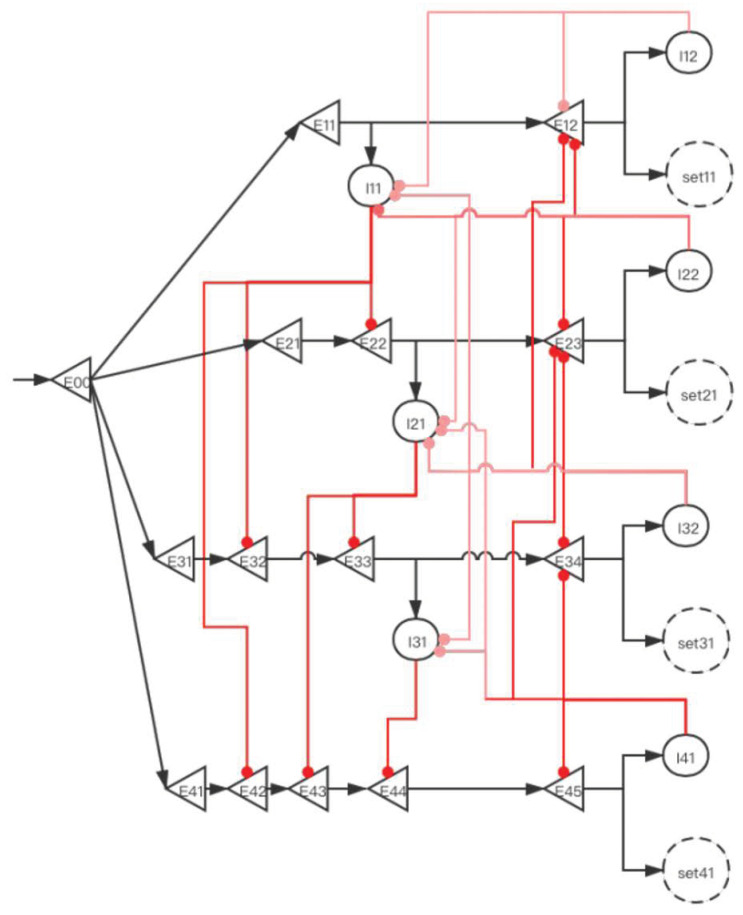
A storage distribution network implemented by pyramidal cells and interneurons. Light red indicates the inhibitory connection of interneurons to interneurons, and dark red indicates the inhibitory connection of interneurons to pyramidal cells. Pyramidal cells are a type of excitatory neurons, so “(E00, E11, E12...)” are used as the abbreviations for pyramidal cells. Interneurons are a type of inhibitory neurons, so “(I11, I12, ...)” are used as the abbreviations for interneurons.“set” in the paper is the same as “SET”. In the absence of interference, we use the FIFO principle to explain the forgetting of working memory. The network fulfills the forgetting of memory over time at the neural network level and reveals the process of the storage of memory and replacement of multiple memory items at the neural network level.

**Figure 7:**
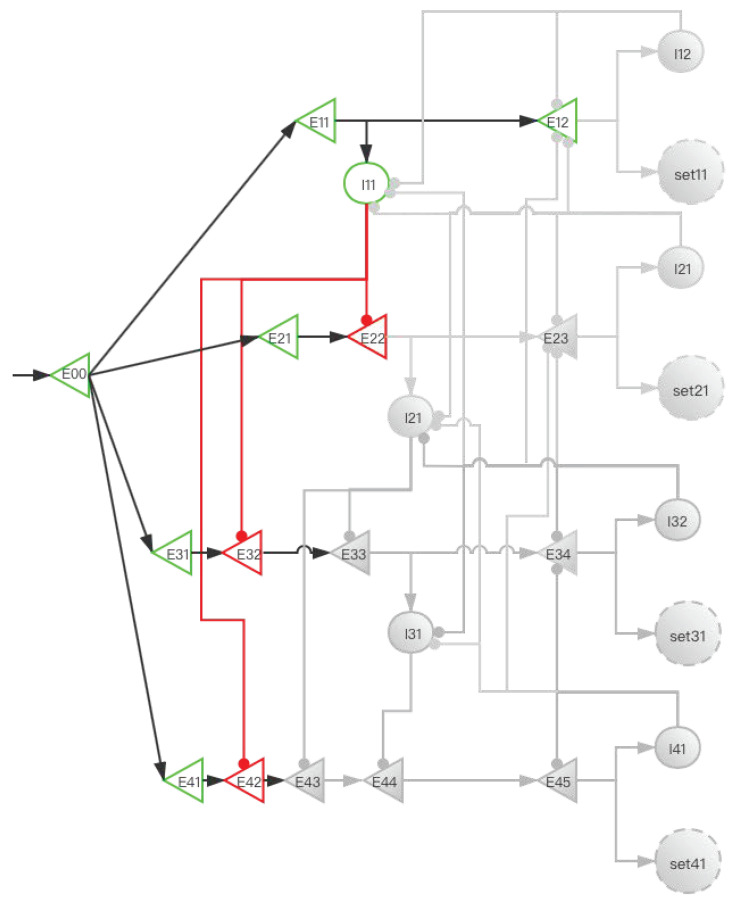
The activity of neurons in the time period from the first signal entering E00 to the activation of E12 (before set11 is activated). After the signal reaches E11, I11 is activated. At this time, E22 also has a signal. However, the inhibitory signal intensity from I11 is greater than the excitatory signal intensity, so E22 is inhibited. Similarly, E32 and E42 are also inhibited; this ensures that the “winner takes all”.

**Figure 8:**
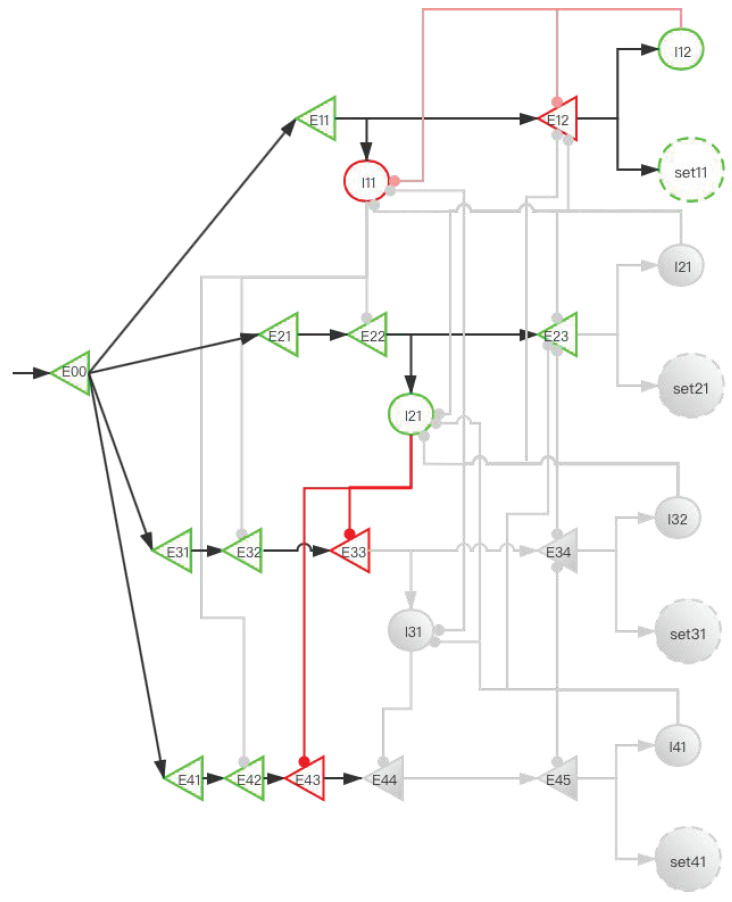
The activity of neurons in the time period from the activation of set11 by the first signal to the activation of E23 by the second signal (before set21 is activated). After the signal enters E12, set11 and I12 are activated, and set11 performs information storage. I12 inhibits I11, which eliminates the influence of I11 on other pyramidal neurons when the second signal enters. The suppression of E12 by I12 increases the difficulty of replacing set11 when other SETs are not activated. After the second signal enters, the E11 branch has been locked, but the latter branch is still open and can be used for storage. E22 activates I21, and I21 is used to inhibit E33 and E43.

**Figure 9:**
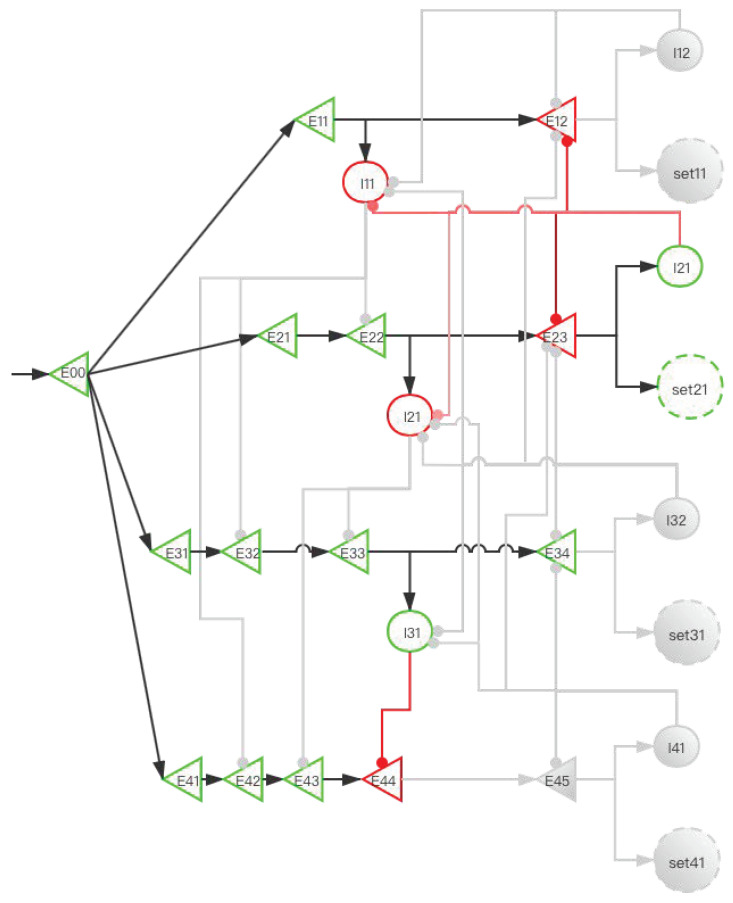
The activity of neurons in the time period from the activation of set21 by the second signal to the activation of E34 by the third signal (before set31 is activated). When E23 is activated, I22 inhibits I11 and I21. This prevents the next signal from entering the E11 branch and E21 branch to affect E31 and E41. The suppression of E12 and E23 by I22 increases the difficulty for set11 and set21 to be activated when set31 and set41 are not activated. After the third signal enters, at this time, the E11 and E21 branches have been locked, but the latter branches are still open and can be used for storage. E33 activates I31, and I31 is used to inhibit E44.

**Figure 10:**
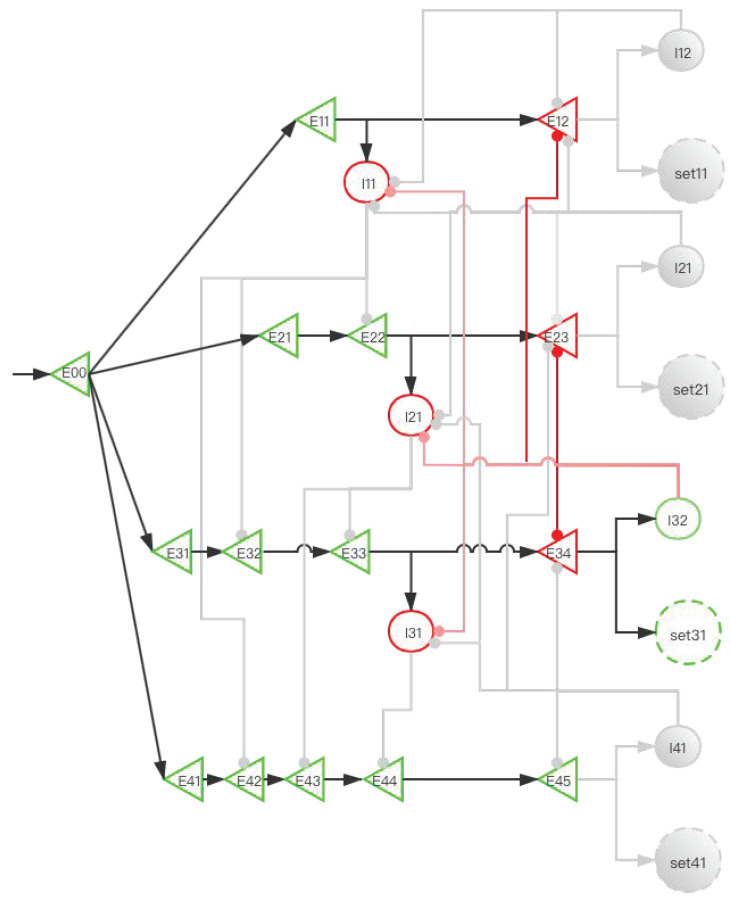
The activity of neurons in the time period from the activation of set31 by the third signal to the activation of E45 by the fourth signal (before set41 is activated). When E34 is activated, I32 inhibits I11, I21, and I31. E12, E23, and E34 have the same effects as Figure 8 and Figure 9.

**Figure 11:**
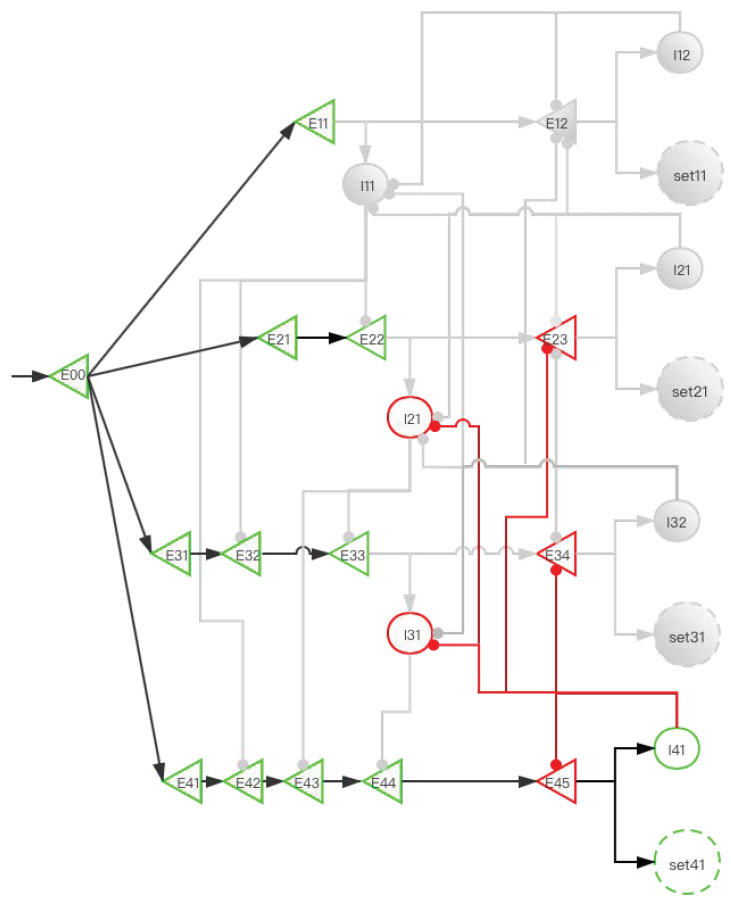
The activity of neurons in the time period from the activation of set41 by the fourth signal to the activation of E11 by the fifth signal (before E12 is activated). After the fourth signal enters, when E45 is activated, I41 inhibits I21, I31, E23, E34, and E45. All neurons in the E11 unit are not inhibited at this time. When the fifth signal is reached, repeat (Figure 7).

The central memory store is limited to three to five meaningful items in young adults [2,3,4]. To facilitate the understanding of SDN (Figure 6), we give the first four simple memory items to stimulate the process from E00 entering the network to the first four memory items being stored in the SET. When four stimulation signals enter the SDN, Figure 7, Figure 8, Figure 9, Figure 10 and Figure 11 show the state changes of excitatory (green) neurons and inhibitory (red) neurons. Gray means that the neurons are in a resting state and do not participate in network activity during this period.

In the network, the memory-storage sub-network can at most store four items with the assistance of SDN. When people want to remember a new item, the human brain will split the item into multiple features and form a control signal for each feature. In our model, one SDN stores one characteristic. Therefore, we try using multiple SDNs to distribute multiple features to complete the process of remembering and forgetting new information. When it is necessary to memorize multiple features, such as the size and color of an object, the differences among the features will be relatively large, and the stimulus intensity received by the neuron will also vary significantly. SDN can distribute new stimuli to the proper SET. In the HPNF model, the new memory can update the old memory. SDN directly selects an idle SET for storage. When we observe an object repeatedly, the same characteristic signal is transmitted to SDN, and SDN selects the SET in which the memory was originally stored. In this way, the firing rate of SET is updated, and its stored memory is enhanced due to memory retelling.

**Figure 12:**
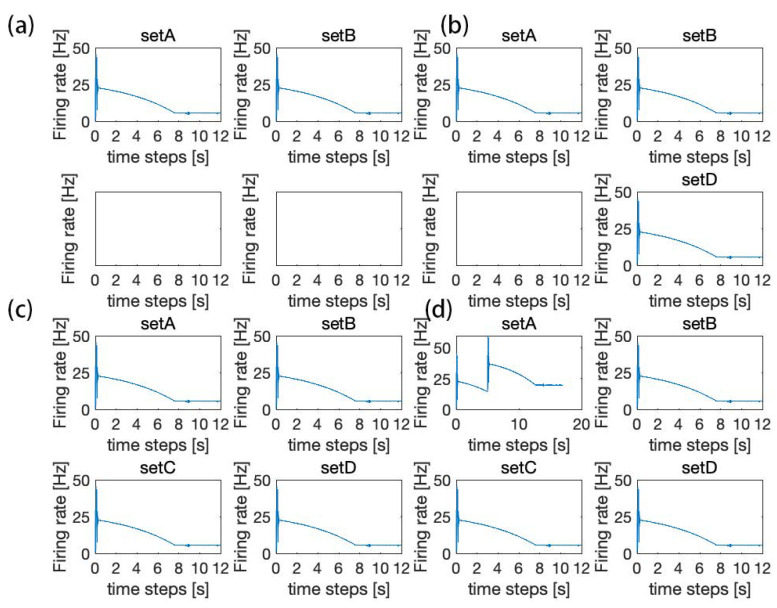
Memory update. If there is a free set (**a**), SDN chooses the free set to store new memory (**b**). If there is no free set (**c**), SDN updates the set that has the lowest firing rate in the SDN (**d**).

Figure 12 shows two possible situations when a new memory item comes in. One is that there is a free set to store the new item (Figure 12a), so the SDN chooses the free set to store memory (Figure 12b). The other situation is that all SETs in the SDN store memory, and set A stores the earliest memory in the selector circuit (Figure 12c). Therefore, set A has the lowest firing rate in the SDN. The SDN deletes the old memory stored in set A and then updates set A with the new memory (Figure 12d).

## 3. Results

In experiments A, B, C, and D, we use electrical signals to simulate the stimulus in human brains, which convert the neural firing rates into more intuitive results. The SDN selects different SETs according to the features of the object. Each SET stores different features of an object, such as the number in Experiment B, the letter in Experiment C, or the color and shape in Experiment D. We use the firing rate of pyramidal cells in SET to represent the memory stored in the cerebral cortex. The firing rate of SET gradually decreases over time, and the memory also becomes blurred. Therefore, the probability of successfully recalling the object‘s features decreases gradually. When the firing rate of SET drops to a certain threshold, it means total forgetting of WM.

Our model duplicated the results of the oculomotor delayed-response task experiment [4], limited-hold memory task experiment [35], encoding and immediate recall of a word list [36], feature association experiment [37], and serial-position effect experiment [38]. The results of the SDN in our experiments obtain the same trend as the above experiments.

### 3.1. Experiment Study A

**Figure 13:**
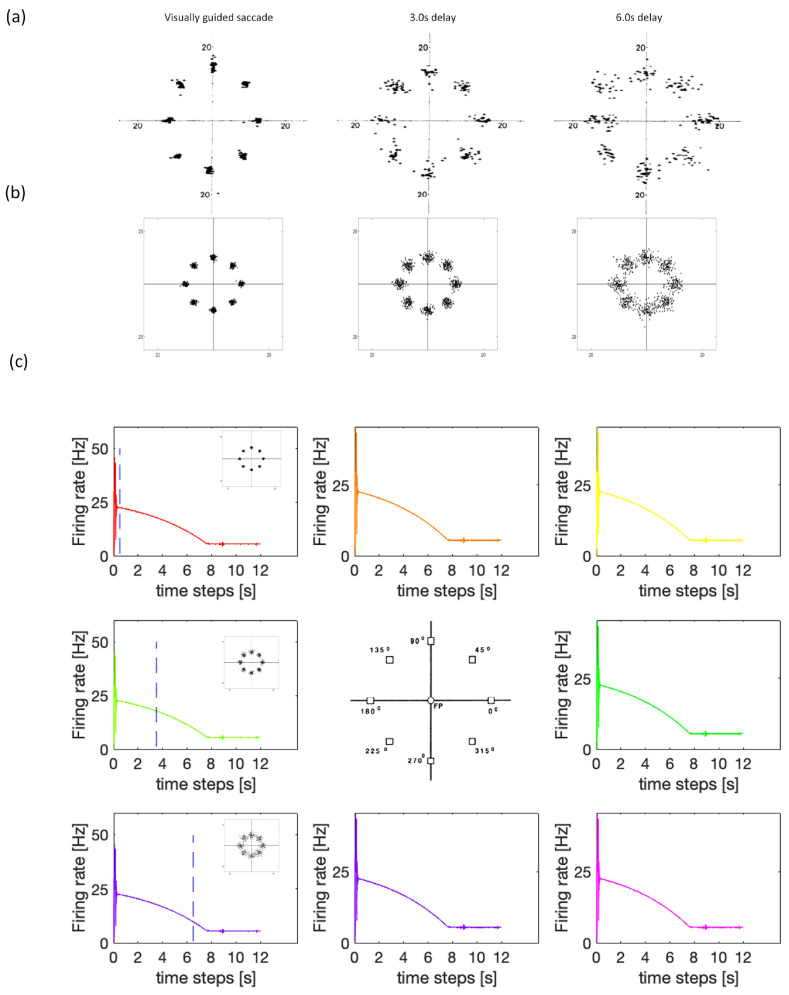
Oculomotor delayed response task experiment on a monkey and the stimulation results of SDN. (**a**) Experimental data from Funahashi et al. using a monkey’s oculomotor delayed-response task. (**b**,**c**) SDN simulation results.

In Experiment A, we compare the SDN’s performance to a monkey’s oculomotor delayed-responses recorded in a 1989 study by Funahashi [4]. The results are shown in Figure 13. At the outset of Funahashi’s trials, each monkey’s eye position was controlled so that it remained gazing at the central spot of a computer monitor. After a brief visual stimulus flashed, there were delay and response periods. During the response period, the central spot disappeared, and then each monkey was allowed to move its eyes freely. The room was dark, and there was nothing shown on the monitor. Funahashi recorded whether the monkey would move its eyes back to the position where the previous visual stimulus had flashed. Different neurons represented different visual positions. Funahashi’s results showed that WM is closely linked to the dorsolateral prefrontal cortex, and many neurons engage in persistent activity to help recall memory.

Figure 13a shows that the monkey’s level of recall accuracy varied for different delay periods. Soon after receiving a stimulus, the monkey could clearly recall the position of the flash. As time went on, the monkey’s memory became increasingly confused, until it finally forgot the position of the flash. We use the SDN to simulate the experiment (Figure 13b,c). The model’s distribution of the firing rates of different components was largest during the response period. The firing rates between different components became closer over time. Figure 13b shows more intuitive results of the firing rate. In this experiment, the HPNF model can sustain the memory’s existence. When time goes on, memories will be forgotten as the firing rate decreases. These results have the same trend as the results of the monkey in Funahashi’s study.

### 3.2. Experiment Study B

**Figure 14:**
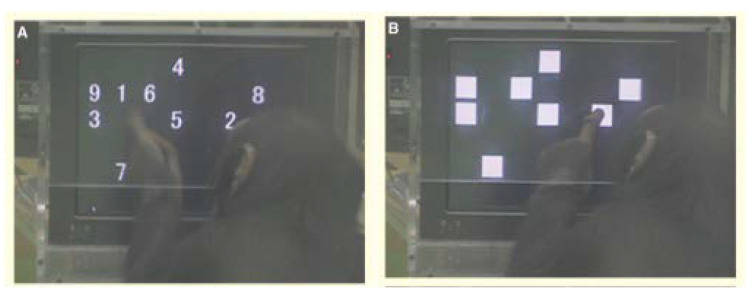
Chimpanzee Ayumu performing the limited-hold memory task. (**A**) Ayumu touches the first number. (**B**) The remaining numbers are covered by white squares.

**Figure 15:**
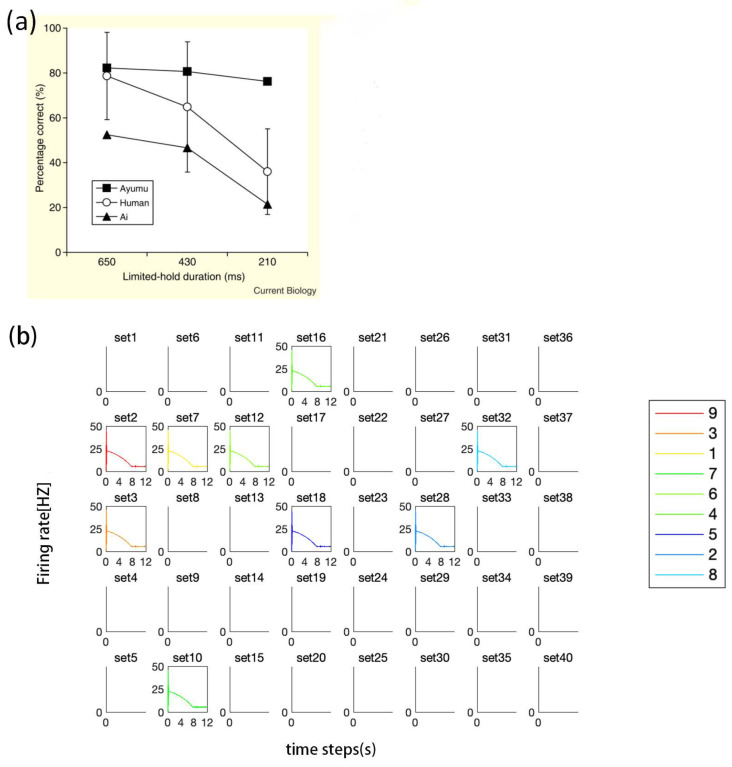
Comparison of SDN and chimpanzees in a limited hold memory task. (**a**) The results of Inoue and Matsuzawa’s chimpanzees in the limited-hold memory task. (**b**) Our model’s simulation during the 210 ms hold time.

We compare the results generated by SDN with the WM study findings of Inoue and Matsuzawa [35], who developed a test called the “limited hold memory task” to compare the WM of chimpanzees and humans. In this test, the numbers 1 to 9 were displayed for a limited time on a touchscreen monitor according to an arrangement shown in Figure 14. The numbers were covered by white squares after the display time was up. Figure 14A shows a chimpanzee, which they had trained to perform well at the task. The chimpanzee performed better than humans in touching the white squares in the original numerical order. The results of our SDN can associate the numbers with their positions. The model’s simulation is consistent with the results of Inoue and Matsuzawa’s experiment (Figure 15).

### 3.3. Experiment Study C

**Figure 16:**
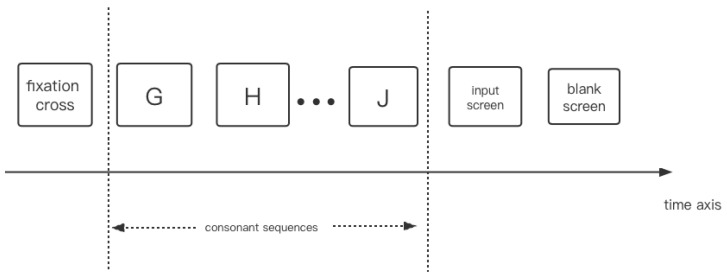
Time course of stimulus presentation. Adapted from [39]. Every character, including the fixation cross, was shown for 200 ms, followed by a blank screen for 800 ms. When ‘Enter’ was pushed, the input screen was replaced by the following blank screen of 1000 ms duration.

**Figure 17:**
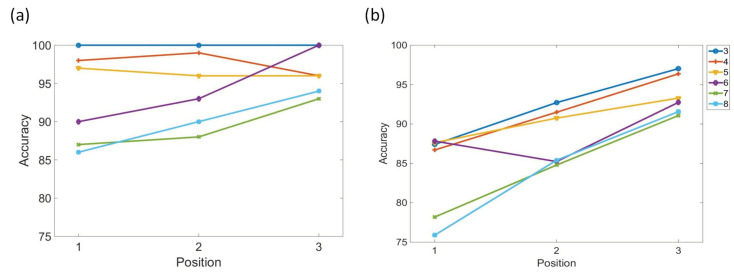
Comparison of the results of SDN and humans in a test devised by Kusak et al. (**a**) The results of Kusak et al.’s experiment. (**b**) Our model simulation results.

We compare the results of SDN to those of humans in a test administered by Kusak et al. [36]. They showed participants 90 consonant sequences constructed with three to eight characters. Each sequence length appeared with the same probability pseudo-random selection. Each character appeared for 200 ms on the screen, and then the screen remained blank for 800 ms. After the last consonant of a sequence, the participants were instructed to reproduce the last three characters in the order of presentation (Figure 16). Figure 17 compares the results of Kusak et al. and the SDN’s simulation. SDN successfully associates the characters with their positions in the sequences, and it also updates old memories with new memories. The results of SDN are consistent with the results of humans in Kusak et al.’s experiment.

### 3.4. Experiment Study D

We compare the results generated by our model to the findings of Wheeler and Treisman’s study [37]. In Wheeler and Treisman’s study, in each trial, the sequence of displays was as follows: A small, black warning cross was presented at the center of the screen for 506 ms. This was followed by a blank screen for 253 ms, which was followed by an initial display of squares flashed for 147 ms, then by a 906-ms blank interval. Finally, there was a test display that remained present until a response was made. The participants indicated by a keypress whether the whole display was the same or different from the initial display. Experiments had four different kinds of changes:

(1) Color only: Participants were told that only the color of items could be changed. In different trials of the test, the same shapes were present, but two items had changed to two new colors. These colors were not previously presented in the initial display.
(2) Shape only: Participants were told that only the shape of items could be changed. In different trials of the test, the same colors were present, but two items had changed to two new shapes. These colors were not previously presented in the initial display.
(3) Either shape or color: Participants were told that either the color or the shape of items could be changed. Half the trials were shape trials in which two squares changed to two new shapes. Half the trials were color trials in which two new colors were presented. These color and shape trials were randomly intermixed with no indication of which type of information would be probed until the test.
(4) Binding shape and color: All the same colors and shapes were presented. However, in different trials, the binding, that is, the relationship between color and shape, changed for two items. In effect, two shapes switched their colors or shapes with each other.

**Figure 18:**
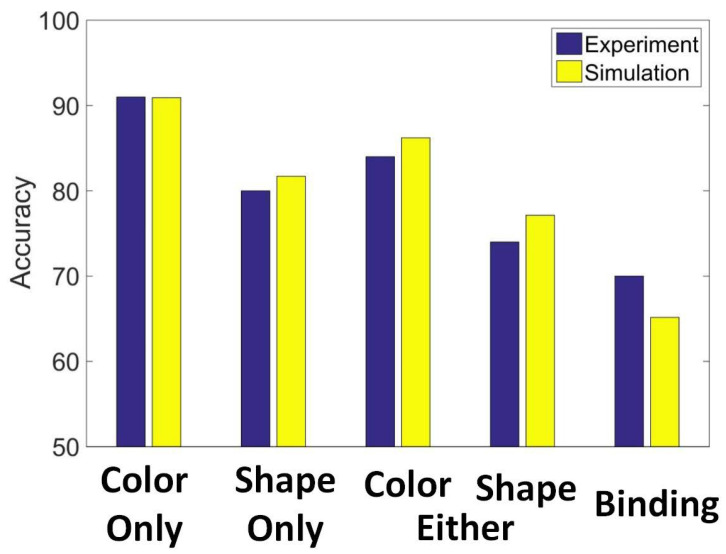
The results of Wheeler and Treisman’s experiment and our model’s simulation results.

Figure 18 shows the results of Wheeler and Treisman’s experiment and our model’s simulation. SDN can associate the specified features in this experiment. It exhibits the same trend as the humans in Wheeler and Treisman’s experiment.

### 3.5. Experiment Study E

**Figure 19:**
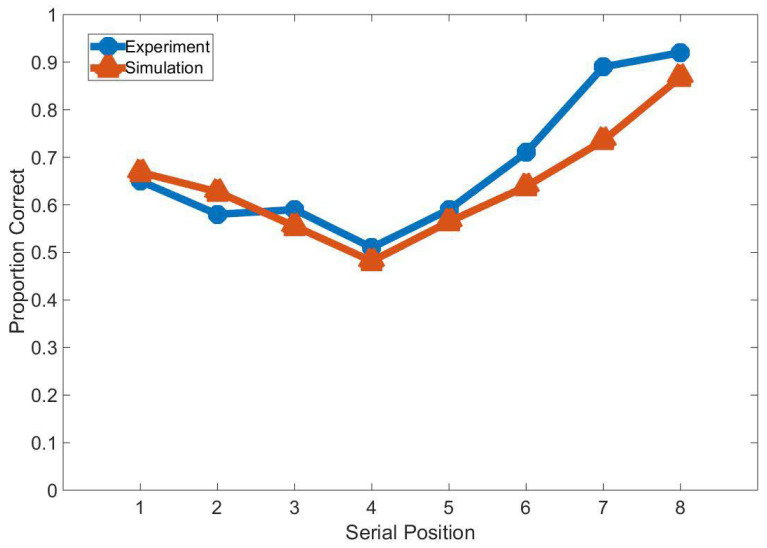
The results of Bhatarah et al.’s experiment and our model’s simulation results.

We compare the results of the SDN with the results of humans in a study by Bhatarah et al. [38]. In the study, eight words were shown to participants for 1 s at 2 s intervals. Participants read each word aloud as it was presented. Then they were asked to recall the words in a 25 s recall period. Figure 19 shows the results of the experiment and SDN’s simulation. SDN can enhance memories through rehearsal in this experiment. SDN’s results had the same trend as the results in Bhatarah et al.’s experiment.

## 4. Conclusions

We propose a new approach to the study of the neural circuits of WM. In the experiments, we use the HPNF model to build a memory-storage sub-network (SET) capable of remembering and forgetting. Compared with the single feedback model, the memory-storage sub-network (SET) is more robust to common disturbance and can adapt well to differences in network parameters among individuals. Additionally, we construct a storage distribution network (SDN), which is used to choose the proper SET and update it according to the firing rate of existing memory and new memory. The SDN limits the maximum amount of memory that can be stored at the same time. The SDN model duplicated the findings of Funahashi et al., Inoue and Matsuzawa, Kusak et al., Wheeler and Treisman, and Bhatarah et al., demonstrating that our proposed approach closely matches many characteristics of WM.

The SET we constructed is a creative modeling work for the self-sustaining of neural information in working memory. Analogous to the storage of memory in computers, SET reveals a topology of neural circuits that may exist in the human brain. At the same time, analogous to the storage distribution and refreshing of memory in computers, SDN also reveals a possible mechanism of memory distribution and forgetting in the process of WM. The models may have a positive impact on the future research on working memory. In addition, recent research shows that WM can be impaired in several psychiatric disorders such as schizophrenia and bipolar disorder and linked to impaired sustained attention [40,41]. The most common cognitive impairment of mild cognitive impairment (MCI) includes episodic memory loss and difficulties in working memory (WM). Research by Sara Aurtenetxe et al. [42] shows that interference can deplete WM, and an optimal WM performance requires effective control of attentional resources between the storage of memory and the incoming stimuli. Our research on memory storage and memory distribution may be potentially helpful for the treatment of some diseases related to memory impairment such as MCI. 

## Figures and Tables

**Table 1:** Comparison between the process of memory management in computer operating system and the process of working memory in the human brain.

Operating System	Human Brain
(1) When the program is running, the operating system needs to allocate memory space in the memory. The memory space takes the block as the basic storage unit. When the process is executing, it applies to the block space in the memory one by one. (2) If there is no target data in the memory, the operating system will schedule the required data block from the hard disk and put it into the memory block. (3) If there is no memory space for storing data, it will use the scheduling algorithm to replace the certain data stored by some previous processes.	(1) The human brain needs to temporarily store each memory item in the process of working memory. The memory items are stored in the hippocampus of the brain [23]. Understanding the interactions between the major cellular constituents of cortical circuits—pyramidal cells and inhibitory neurons—is considered a necessary step in unraveling the cellular mechanisms subserving working memory mechanisms and, ultimately, cognitive processes [24]. (2) In the process of working memory in the human brain, little is known about the structure of a single memory item stored in the hippocampus at the neural circuit level. (3) It has recently been proposed that working memory might better be conceptualized as a limited resource that is distributed flexibly among all items to be stored in memory [25]. At the neural circuit level, little is known about how the hippocampus distributes multiple memory items to multiple neurons (groups).

## Data Availability

Not applicable.
